# The cytokine-driven regulation of secretoglobins in normal human upper airway and their expression, particularly that of uteroglobin-related protein 1, in chronic rhinosinusitis

**DOI:** 10.1186/1465-9921-12-28

**Published:** 2011-03-08

**Authors:** Xiang Lu, Nan Wang, Xiao-Bo Long, Xue-Jun You, Yong-Hua Cui, Zheng Liu

**Affiliations:** 1 Department of Otolaryngology-Head and Neck Surgery, Tongji Hospital, Tongji Medical College, Huazhong University of Science and Technology, Wuhan, China

## Abstract

**Background:**

The involvement of secretoglobins (SCGBs) other than SCGB1A1 (Clara cell 10-kDa protein, CC10) in human airway diseases remains unexplored. Among those SCGBs, SCGB3A2 (uteroglobin-related protein 1, UGRP1) is particularly interesting, given its structure and function similarities with SCGB1A1 (CC10). The aim of this study was to investigate the expression regulation of SCGBs other than SCGB1A1 (CC10) in human upper airway, and their potential involvement, particularly that of SCGB3A2 (UGRP1), in chronic rhinosinusitis (CRS) with nasal polyps (CRSwNP) and without nasal polyps (CRSsNP).

**Methods:**

Eight SCGB family members including SCGB3A2 (UGRP1), SCGB1C1 (ligand binding protein RYD5), SCGB1D1 (lipophilin A), SCGB1D2 (lipophilin B), SCGB1D4 (interferon-γ inducible SCGB), SCGB2A1 (mammaglobin 2), SCGB2A2 (mammaglobin 1), and SCGB3A1 (uteroglobin-related protein 2) were studied. The regulation of SCGBs mRNA expression in normal nasal mucosa by proinflammatory, Th1, and Th2 cytokines was studied through nasal explant culture. SCGBs mRNA expression levels in CRSsNP and CRSwNP patients and controls were compared. The mRNA levels were detected by means of quantitative reverse transcriptase-polymerase chain reaction. The protein expression of SCGB3A2 (UGRP1) was analyzed using immunohistochemistry.

**Results:**

The expression of SCGBs except SCGB1D2 (lipophilin B) could be found in upper airway and be differentially regulated by different cytokines. SCGB3A2 (UGRP1) mRNA expression was induced by Th1 cytokine, but suppressed by proinflammatory and Th2 cytokines. SCGBs mRNA expression was altered in CRS; particularly, SCGB3A2 (UGRP1) protein and mRNA expression was markedly decreased in both CRSsNP and CRSwNP and its protein levels inversely correlated with the number of total infiltrating cells, preoperative sinonasal CT scores, and postoperative endoscopy and symptom scores.

**Conclusion:**

SCGBs except SCGB1D2 (lipophilin B) are expressed in human upper airway and their expression can be differentially regulated by inflammatory cytokines. SCGBs mRNA expression is altered in CRS. Reduced production of UGRP1, which is likely due, at least in part, to a local cytokine environment, may contribute to the hyper-inflammation in CRS and correlates with response to surgery.

## Background

Secretoglobin (SCGB) superfamily is a group of small, secreted, dimeric proteins [1-5]. Our knowledge of the SCGB superfamily is rapidly expanding with the discovery of many new human genes. At present, nine members of this superfamily have been identified in humans, which includes SCGB1A1 (Clara cell 10-kDa protein, CC10), SCGB1C1 (ligand binding protein RYD5, RYD5), SCGB1D1 (lipophilin A, LIPA), SCGB1D2 (lipophilin B, LIPB), SCGB1D4 (interferon-γ inducible SCGB, IIS), SCGB2A1 (mammaglobin 2, MGB2), SCGB2A2 (mammaglobin 1, MGB1), SCGB3A1 (uteroglobin-related protein 2, UGRP2), and SCGB3A2 (uteroglobin-related protein 1, UGRP1) [1-5]. Although the expression of SCGBs has generally been associated with secretory epithelia, such as in the mammary gland and prostate, there have been only few reports of their expression and regulation in human and rodent airways [1-5].

The pathophysiological functions of SCGBs are poorly understood. Limited studies indicate that some SCGBs are associated with malignancies, such as SCGB2A2 (MGB1), SCGB2A1 (MGB2), and SCGB1D1 (LIPA) [1,2], and some are involved in immune responses, such as SCGB1A1 (CC10), SCGB1D4 (IIS), SCGB3A1 (UGRP2), and SCGB3A2 (UGRP1) [3-7]. CC10 is a prototypical member of SCGB superfamily with anti-inflammatory and immunomodulatory effects. Previous studies from us and others have implicated the diminished expression of CC10 in the pathogenesis of inflammatory upper and lower airway diseases including chronic rhinosinusitis (CRS) and asthma [5-8]. However, as to other SCGBs, whether they are also involved in airway diseases has been rarely studied.

Among SCGB family members, SCGB3A2 (UGRP1) is particularly interesting. SCGB3A2 (UGRP1) possesses significant amino acid sequence similarity to CC10 (SCGB1A1) [4,9]. Intranasal administration of recombinant adenovirus expressing SCGB3A2 (UGRP1) suppresses the allergen-induced eosinophilic lung inflammation in a mouse model, indicating that SCGB3A2 (UGRP1) may possess a similar anti-inflammatory function as SCGB1A1 (CC10) [9]. Genetic analysis has demonstrated that a single nucleotide polymorphism (SNP) (G/A) at -112bp of the human SCGB3A2 (UGRP1) gene promoter is associated with an increased risk of asthma in a Japanese population [10,11]. Nevertheless, the expression and the role of SCGB3A2 (UGRP1) in human airway diseases remain largely unknown.

CRS is a complex and heterogeneous syndrome and typically classified into CRS with nasal polyps (CRSwNP) and CRS without nasal polyps (CRSsNP) [12,13]. To date, although the etiology and the pathogenesis of CRS remain a matter of vigorous debate, the imbalance between pro- and anti-inflammatory responses is believed to initiate and sustain the inflammation exacerbation in CRS [5,7,12,13]. Therefore, indentifying the factors influencing this imbalance would provide new insights into the pathogenesis of CRS.

The purposes of the present study were: (1) to examine the regulation of SCGBs mRNA expression other than SCGB1A1 (CC10) in human normal nasal mucosa by inflammatory cytokines; (2) to compare the expression levels of SCGBs in normal controls and CRSwNP and CRSsNP patients; and (3) to further study the SCGB3A2 (UGRP1) protein expression and its significance in CRS.

## Methods

### Subjects

This study was approved by the ethical committee of Tongji Medical College of Huazhong University of Science and Technology and conducted with written informed consent from patients.

1. SCGBs expression regulation in *ex vivo *cultured normal nasal mucosa study: This study population comprised 34 patients undergoing septal surgery and/or turbinectomy because of nasal obstruction. None had a history of persistent mucopurulent drainage, allergic rhinitis, or sinus disease. Inferior turbinate mucosal samples were used for nasal explant culture [5].

2. SCGBs expression in CRS study: Twenty patients with CRSsNP and 20 patients with CRSwNP who had bilateral CRS were recruited. The diagnosis of CRSsNP and CRSwNP was made according to the current European EAACI Position Paper on Rhinosinusitis and Nasal Polyps and American guideline [12,13]. Diseased ethmoid sinus mucosa from the most hypertrophied and hyperemic regions and NP tissues from the apex region of polyps were collected during surgery. As controls, inferior turbinate mucosal samples were taken during surgery from 16 patients undergoing septoplasty and/or turbinectomy because of nasal obstruction and not having any sinus disease or allergic rhinitis. Surgical samples were processed for histology, reverse transcriptase-polymerase chain reaction (RT-PCR), and immunohistochemistry study.

In our study, subjects who had an antrochoanal polyp, cystic fibrosis, fungal sinusitis, or primary ciliary dyskinesia were excluded. None of the patients had an acute upper respiratory infection in the four weeks before the operation. The atopic status was evaluated by skin prick test to a standard panel of aeroallergens. The diagnosis of asthma and aspirin sensitivity was based on history and physician diagnosis. Oral glucocorticoid and intranasal steroid sprays were discontinued at least 3 months and 1 month before surgery, respectively. None had received antileukotrienes and immunotherapy. All patients and controls were Han Chinese from central China.

Clinical data of patients are summarized in Table 1.

**Table 1:** Clinical data of patients enrolled in SCGBs expression regulation study and SCGBs expression in CRS study

	SCGBs expression in CRS study			SCGBs expression regulation study
		
	Control	CRSsNP	CRSwNP	
Subject, n	16	20	20	34
Sex, male, n (%)	10 (62.5)	11 (55)	13 (65)	20 (58.8)
Age (years), mean ± SD	30.7 ± 11.4	35.5 ± 11.0	34.4 ± 12.7	31.7 ± 12.0
Patients with asthma, n (%)	0 (0)	2 (10)	7 (35)	0 (0)
Patients with positive skin prick test results, n (%)	0 (0)	5 (25)	8 (40)	0 (0)
Patients with aspirin sensitivity, n (%)	0 (0)	0 (0)	0 (0)	0 (0)

SCGBs, secretoglobins; CRSsNP, chronic rhinosinusitis without nasal polyps; CRSwNP, chronic rhinosinusitis with nasal polyps.

### Assessment of CRS clinical severity

For CRS patients, preoperative coronal CT scans through paranasal sinuses were obtained and scored using the Lund-Mackay system, as previously described [14]. Symptom evaluation and endoscopic examination were taken before and 12 months after the surgery. A symptom questionnaire based on a visual analog score (VAS) of 0 to 10 according to severity was used. A total VAS score was calculated based on the sum of five VAS symptom domains, including nasal blockage, headache, facial pain, alteration of sense of smell, and nasal discharge [5]. In addition, patients were asked to rate his/her overall burden of CRS symptoms [5]. Endoscopy physical findings were scored according to Lanza and Kennedy [15].

### Nasal explant culture

Normal inferior turbinate mucosal tissues were obtained during surgery and immediately cut into multiple fragments of approximately 6 mm^3^. One was processed for histologic evaluation and others were used for tissue culture. Sections of tissue were placed on 0.4 mm-well inserts (Millipore Corp., Billerica, MA, USA) in 2 mL of Dulbecco's modified Eagle's medium/F-12 supplemented with 2 mM L-glutamine, 100 U/mL penicillin, and 100 mg/mL streptomycin (Invitrogen, Grand Island, NY, USA). The tissue was oriented with the epithelium being exposed to the air, forming an air-liquid interface to mimic the *in vivo *situation [6,16,17]. For dose response experiments, the tissues were incubated in the presence of TNF-α (1, 10, and 100 ng/ml), IL-1β (1, 10, and 100 ng/ml), INF-γ (0.1, 1, and 10 ng/ml), IL-4 (1, 10, and 100 ng/ml), or IL-13 (1, 10, and 100 ng/ml) for 12 h. These cytokines were purchased from R&D Systems (Minneapolis, MN, USA). For time course experiments, the tissues were incubated with TNF-α (20 ng/ml), IL-1β (20 ng/ml), IL-4 (20 ng/ml), IL-13 (20 ng/ml), or INF-γ (1 ng/ml) for various time durations between 4 and 24 h. The tissues were cultured at 37˚C with 5% CO_2 _in humidified air.

### Quantitative RT-PCR

Freshly obtained tissues were immediately snap frozen in liquid nitrogen. RNA was extracted and cDNA was reverse transcribe as previously described [16]. The PCR assays for the members of SCGB family were performed using the SYBR Premix Ex Taq kit [TaKaRa Biotechnology (Dalian), Dalian, China] with appropriate primers constructed from published sequences (Table 2) as mentioned elsewhere [16]. GAPDH was used as a housekeeping gene for normalization and 'no template' sample was used as a negative control. Relative gene expression was calculated by using the comparative CT method [16]. An inferior turbinate sample was used as a calibrator in SCGBs expression in CRS study, whereas respective control tissues without any cytokine stimulation were employed as calibrators for SCGBs expression regulation study. The identity of PCR product was confirmed by DNA sequencing.

**Table 2:** Primers used for quantitative PCR analysis of SCGB genes expression

Gene	Accession number	Primers sequence	Annealing temperature (˚C)
SCGB3A2 (UGRP1)	NM_054023	forward,5'-CGGAATTCCCCAGATAACTGTCA-3'	60
		reverse, 5'-ACATCTAGACACCAAGTGTGATAGC-3'	
SCGB1C1 (RYD5)	NM_145651	forward, 5'-TGGCCCTCACCCTGTTCTGCATCT-3'	60
		reverse, 5'-CACCGTGCTGACTGCCCAGCAGTT-3'	
SCGB1D1 (LIPA)	NM_006552	forward, 5'-CAGTGGTCTGCCAAGCTCTTGG-3'	60
		reverse, 5'- CATAGGCCATCGTATCCACGC-3'	
SCGB1D2 (LIPB)	NM_006551	forward, 5'-CCTCTGTTCAAGTTAAGTC-3'	58
		reverse, 5'-CCGCAATGAGGCTTCGTTTGG-3'	
SCGB1D4 (IIS)	NM_206998	forward, 5'-CTCACAGCCGAATAAGCCACC-3'	55
		reverse, 5'-GTGCAGGGCAAGTGATTTATTAAAGC-3'	
SCGB2A1 (MGB2)	NM_002407	forward, 5'-CTCCTGGAGGACATGGTTG-3'	60
		reverse, 5'-CTATGTGACTGGTTGAGG-3'	
SCGB2A2 (MGB1)	NM_002411	forward, 5'-GACAATGCCACTACAAATGCC-3'	60
		reverse, 5'-CATTGCTCAGAGTTTCATCCG-3'	
SCGB3A1 (UGRP2)	NM_052863	forward, 5'- CGGAATTCCCCCGCGCCATGAAGCTC-3'	66
		reverse, 5'- ACATCTAGAGCCAAACACTGTCAGG-3'.	
GAPDH	NM_002046	forward,5'-GAAGGTGAAGGTCGGAGTC-3'	60
		reverse, 5'-GGAAGATGGTGATGGGATT-3'	

SCGB, secretoglobin; UGRP1, uteroglobin-related protein 1; RYD5, ligand binding protein RYD5; LIPA, lipophilin A; LIPB, lipophilin B; IIS, interferon-γ inducible secretoglobin; MGB2, mammaglobin 2; MGB1, mammaglobin 1; UGRP2, uteroglobin-related protein 2.

### Routine staining, SCGB3A2 (UGRP1) immunohistochemistry, and quantification

Paraffin sections (4 μm) were stained with hematoxylin and eosin. The number of inflammatory cells per high-power filed (HP) was determined by counting 10 randomly selected fields in a blinded fashion at 400 × magnification by 2 independent physician who were blind to the clinical data. The difference in counting results between 2 independent investigators was less than 10%. In case of disagreement (the two counts differed by > 10%), a consensus was reached by reviewing the specimen at a multihead microscope by our research team.

As to immunohistochemical staining of SCGB3A2 (UGRP1) protein, the sections were stained with goat anti-SCGB3A2 (UGRP1) antibody (1:100; R&D Systems). SCGB3A2 (UGRP1) was detected using the streptavidin-peroxidase complex method with a histostain-plus kit (Zhongshan Golden Bridge Biotechnology, Beijing, China) as previously described [16]. Color development was achieved with 3', 3'-diaminobenzidine, which rendered positive cells brown. A species-matched antibody was used as a negative control. Quantitative measurement of SCGB3A2 (UGRP1) protein expression was analyzed using the HPIAS-1000 automated image analysis system as described elsewhere [16]. Ten microscopic fields were randomly selected from each slide under × 400 magnification. Results were presented as 1/gray scores, which positively correlate with the intensity of immunoreactivity [16].

### Statistical analysis

Results are presented as mean ± SD, or in box-and-whisker plots. Paired sets of data were compared with Mann-Whitney *U*-test. The Spearman test was used to determine correlations. Paired *t*-test was used in tissue culture data analysis. Data analyses were performed by using SPSS for Windows (SPSS Inc., Chicago, IL, USA). The level of significance was considered at a *P *value of less than 0.05.

## Results

### The cytokine-driven regulation of SCGBs mRNA expression in normal nasal mucosa

**Figure 1:**
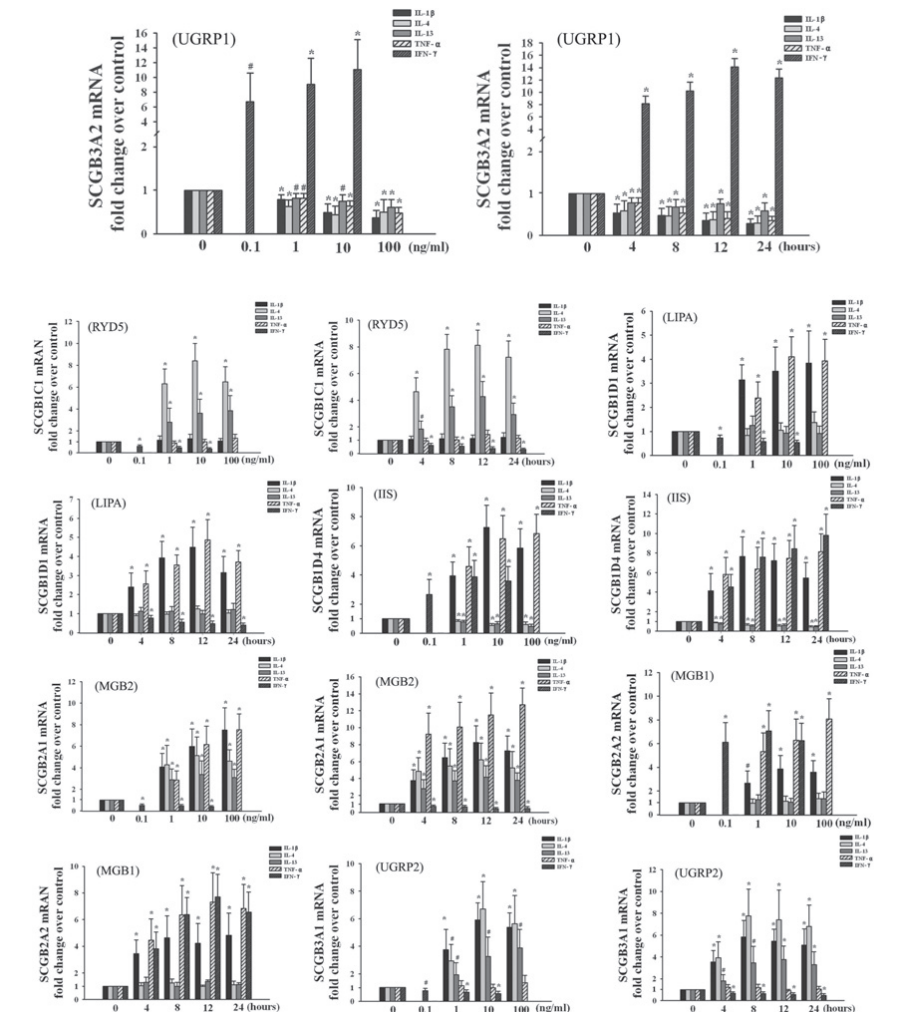
**The effect of cytokines on secretoglobins (SCGBs) mRNA expression in *ex vivo *cultured normal nasal mucosa**. For dose response experiments, the normal inferior turbinate tissues were incubated in the presence of TNF-α (1, 10, and 100 ng/ml), IL-1β (1, 10, and 100 ng/ml), INF-γ (0.1, 1, and 10 ng/ml), IL-4 (1, 10, and 100 ng/ml), or IL-13 (1, 10, and 100 ng/ml) for 12 h. For time course experiments, the tissues were incubated with TNF-α (20 ng/ml), IL-1β (20 ng/ml), IL-4 (20 ng/ml), IL-13 (20 ng/ml), or INF-γ (1 ng/ml) for various time durations between 4 and 24 h. *n *= 6. Results are expressed as mean ± SD. ^#^*P *< 0.05 and *P < 0.01 compared with untreated mucosa.

Since our previous studies have thoroughly investigated the expression and regulation of SCGB1A1 (CC10) in upper airways [5-7], we only detected the mRNA expression profiles of other eight SCGBs in nasal mucosa in the current study. Their mRNA expression could be found in normal nasal mucosa except SCGB1D2 (LIPB), whose expression could not be detected even after 40 cycles of PCRs. As illustrated in Figure 1, IL-1β, TNF-α, IL-4, and IL-13 inhibited, whereas IFN-γ promoted, SCGB3A2 (UGRP1) mRNA expression in normal nasal mucosa. On the contrary, IL-1β, TNF-α, IL-4, and IL-13 enhanced, whereas IFN-γ suppressed, SCGB2A1 (MGB2) mRNA expression. Regarding SCGB1C1 (RYD5), IFN-γ down-regulated and IL-4 and IL-13 up-regulated its expression; however, no significant effect was observed for IL-1β and TNF-α. Nevertheless, as to SCGB1D4 (IIS), IL-1β, TNF-α, and IFN-γ increased, but IL-4 and IL-13 decreased, its expression. With reference to SCGB1D1 (LIPA), its expression could be induced by IL-1β and TNF-α but depleted by IFN-γ, and no significant effect was demonstrated for IL-4 and IL-13. After stimulation with IL-1β, TNF-α, and IFN-γ, the expression of SCGB2A2 (MGB1) was induced markedly; however, no significant effect was discovered for IL-4 and IL-13. Finally, for SCGB3A1 (UGRP2), IL-1β, IL-4, and IL-13 enhanced whereas IFN-γ diminished its expression, and TNF-α exerted no significant influence.

### The mRNA expression of SCGBs in sinonasal mucosa from controls and CRS patients

**Figure 2:**
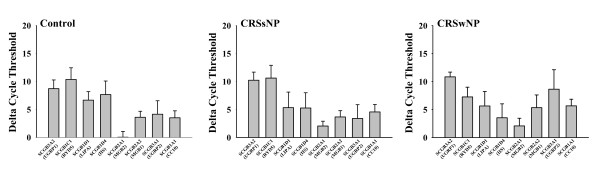
**Relative mRNA expression levels of different SCGBs in sinonasal mucosa**. The relative expression levels are expressed as ΔCT (ΔCT = the difference in threshold cycles for target and GAPDH). This is a direct reflection of amount of input of target mRNA, and a change of CT value of 1 unit is equal to a doubling, or halving, of the level of target mRNA. The higher the ΔCT value, the lower the level of target mRNA. In control group, *n *= 16; in chronic rhinosinusitis without nasal polyps (CRSsNP) group, *n *= 20; and in chronic rhinosinusitis with nasal polyps (CRSwNP) group, *n *= 20. Results are presented as mean ± SD.

The relative expression levels of different SCGBs in sinonasal mucosa from controls, CRSsNP, and CRSwNP patients are presented in Figure 2. The data shown in Figure 2 are expressed as ΔCT (ΔCT = the difference in threshold cycles for target and GAPDH). This is a direct reflection of amount of input of target mRNA, and a change of CT value of 1 unit is equal to a doubling, or halving, of the level of target mRNA. The higher the ΔCT value, the lower the level of target mRNA. SCGB2A1 (MGB2) had the highest expression levels in sinonasal mucosa from both controls and CRS patients. The relative high expression levels were detected for SCGB2A2 (MGB1), SCGB 1A1 (CC10), and SCGB3A1 (UGRP2) in sinonasal mucosa from controls and CRSsNP patients. In CRSwNP patients, relative high abundance of transcripts was found for SCGB1D4 (IIS).

**Figure 3:**
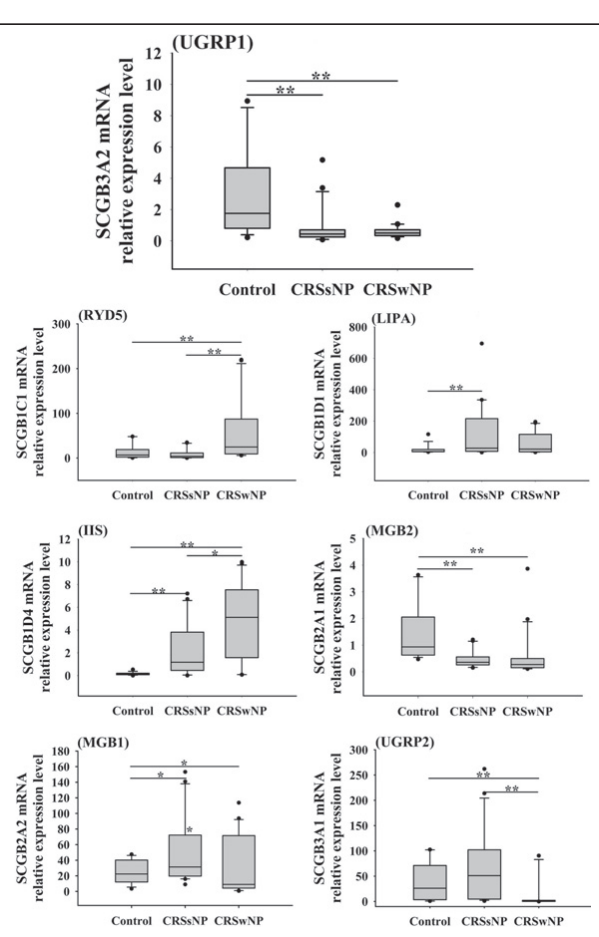
**The mRNA expression profiles of secretoglobins (SCGBs) in chronic rhinosinusitis (CRS)**. In control group, *n *= 16; in CRS without nasal polyps (CRSsNP) group, *n *= 20; and in CRS with nasal polyps (CRSwNP) group, *n *= 20. Data are presented in box-and-whisker plots. *P < 0.05; **P < 0.01.

As shown in Figure 3, compared with controls, SCGB3A2 (UGRP1) and SCGB2A1 (MGB2) mRNA expression was significantly down-regulated, whereas SCGB2A2 (MGB1) mRNA expression was markedly up-regulated, in both CRSsNP and CRSwNP with no significant difference between CRSsNP and CRSwNP. With regard to SCGB1D4 (IIS), its expression was increased in both CRSsNP and CRSwNP with a more prominent increase in CRSwNP. The expression of SCGB1C1 (RYD5) was only increased, whereas the expression of SCGB3A1 (UGRP2) was only decreased, in CRSwNP, and there was a significant difference between CRSsNP and CRSwNP. As to SCGB1D1 (LIPA), its expression was only enhanced in CRSsNP in comparison with control. We did not detect the SCGB1D2 (LIPB) mRNA expression in CRS either. Since the sample size of atopic or asthmatic patients was not large enough in either CRSsNP or CRSwNP group, we did not compare the difference in SCGBs expression between atopic and non-atopic patients, and asthmatic and non-asthmatic patients.

### SCGB3A2 (UGRP1) protein expression and its correlation with inflammatory cells infiltration and clinical features in CRS

**Figure 4:**
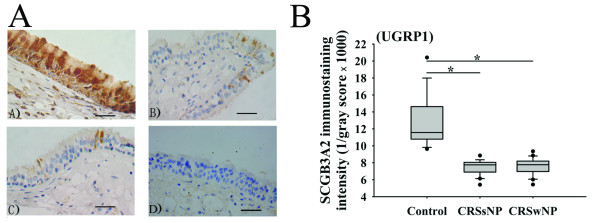
**The protein expression of secretoglobin 3A2 (SCGB3A2) (uteroglobin-related protein 1, UGRP1) in chronic rhinosinusitis (CRS)**. A: A)-C) Representative photomicrographs of SCGB3A2 (UGRP1) immunohistochemical staining of sinonasal tissue sections from A) control, B) CRS without nasal polyps (CRSsNP), and C) CRS with nasal polyps (CRSwNP); D) A photomicrograph of a section stained with a control antibody. Scale bars, 50 μm. B: Quantification of protein expression levels of SCGB3A2 (UGRP1) examined by immunohistochemistry. Immunostaining intensity was analyzed using the automated image analysis system. Results are presented as 1/gray scores, which positively correlate with the intensity of immunoreactivity. In control group, *n *= 16; in CRSsNP group, *n *= 20; and in CRS CRSwNP group, *n *= 20. Data are presented in box-and-whisker plots. *P < 0.05.

We further studied the protein expression of SCGB3A2 (UGRP1) in CRS. Immunohistochemical staining showed that SCGB3A2 (UGRP1) was mainly expressed by epithelial cells (Figure 4A). Confirming the mRNA data, we found that SCGB3A2 (UGRP1) protein expression was significantly decreased in both CRSsNP and CRSwNP in comparison with controls and no significant difference was found between CRSsNP and CRSwNP (Figure 4B). The inflammatory cells infiltration was evaluated by hematoxylin and eosin staining. The numbers of eosinophils, mononuclear cells, and total infiltrating cells in the CRSsNP and CRSwNP group were listed as following: 2.85 ± 1.73 vs. 7.15 ± 3.67 cells/HP (*P *< 0.01), 32.30 ± 10.85 vs. 40.60 ± 13.80 cells/HP (*P *> 0.05), and 67.05 ± 18.06 vs. 76.25 ± 15.07 cells/HP (*P *> 0.05), respectively. Analyzing the relationship between SCGB3A2 (UGRP1) staining intensity and the number of inflammatory cells, we found that SCGB3A2 (UGRP1) staining intensity inversely correlated with the number of total infiltrating cells (*r *= -0.485 and -0.558 in the CRSsNP and CRSwNP group, respectively; *P *< 0.05 for both), but did not correlate with the number of eosinophils or mononuclear cells.

A significant negative correlation was found between SCGB3A2 (UGRP1) staining intensity and pre-operative CT scores (*r *= -0.54 and *r *= -0.45 in CRSsNP and CRSwNP group, respectively; *P *< 0.05 for both), but not pre-operative symptom scores. After surgery, eighteen CRSsNP patients and 19 CRSwNP patients completed 1-year follow-up records. We found that SCGB3A2 (UGRP1) staining intensity inversely correlated with post-operative endoscopy scores (*r *= -0.50 and *P *< 0.05 in CRSsNP; *r *= -0.58 and *P *< 0.01 in CRSwNP), overall VAS symptom scores (*r *= -0.54 and *r *= -0.47 in CRSsNP and CRSwNP group, respectively; *P *< 0.05 for both), and total VAS symptom scores (*r *= -0.53 and *r *= -0.46 in CRSsNP and CRSwNP group, respectively; *P *< 0.05 for both). The data of CT, VAS, and endoscopy scores are provided in Table 3.

**Table 3:** Disease severity assessment

	CRSsNP	CRSwNP
Pre-operation		
CT scores	8.40 ± 4.21	14.90 ± 5.69
Endoscopy scores	4.55 ± 1.90	7.05 ± 2.04
Overall VAS scores	6.15 ± 1.39	7.15 ± 1.48
Total VAS scores	23.35 ± 5.09	23.90 ± 4.78
Post-operation		
Endoscopy scores	1.61 ± 1.33	3.05 ± 2.22
Overall VAS scores	2.33 ± 2.28	3.11 ± 1.73
Total VAS scores	5.72 ± 5.44	7.68 ± 6.29

CRSsNP, chronic rhinosinusitis without nasal polyps; CRSwNP, chronic rhinosinusitis with nasal polyps.

Pre-operation, in CRSsNP group, *n *= 20; and in CRSwNP group, *n *= 20

Post-operation, in CRSsNP group, *n *= 18; and in CRSwNP group, *n *= 19

## Discussion

In the present study, extending our previous finding of SCGB1A1 (CC10) [5,6], we demonstrated the cytokine-driven expression regulation of SCGB superfamily members in human upper airways and their overall expression profile in CRS for the first time. We detected the mRNA expression of all SCGBs except SCGB1D2 (LIPB) in sinonasal mucosa. The expression of SCGB1D2 (LIPB) is also absent in normal lung tissues [1], suggesting that this SCGB may be not important for airway functions. We confirmed the mRNA expression of SCGB2A1 (MGB2) in nasal mucosa, which has been demonstrated by previous gene array studies [6,18]. Our unpublished data indicate that SCGB2A1 (MGB2) is expressed by submucosal glands in sinonasal mucosa. Previous investigations have also indentified some of different SCGBs in other parts of the human respiratory tract, such as SCGB3A2 (UGRP1) and SCGB3A1 (UGRP2) in the epithelium of lung, and SCGB2A2 (MGB1) in trachea [1,19,20]. Our current study demonstrated that in sinonasal mucosa SCGB3A2 (UGRP1) was also mainly produced by epithelial cells. By contrast, SCGB1C1 (RYD5) and SCGB1D4 (IIS) have, to the present author's knowledge, not been demonstrated in human respiratory airway mucosa until now, although SCGB1C1 (RYD5) and SCGB1D4 (IIS) have been detected in Bowman's glands of rat olfactory mucosa and human lymphoid tissues and cells, respectively [3,21]. The cellular locations of SCGB1C1 (RYD5) and SCGB1D4 (IIS) in sinonasal mucosa are unclear. Due to the lack of commercially available antibodies, further *in situ *hybridization study may be helpful in clarifying their cellular locations. The expression regulation of SCGBs has rarely been studied. In airway inflammation, proinflammatory, and Th1 and Th2 relevant cytokines play a crucial role. The increased expression of IL-1β, TNF-α, INF-γ, and IL-4 has been demonstrated in CRS, which may be involved in the inflammation perpetuation and exaggeration [5]. Therefore, in this study, SCGBs expression regulation by these cytokines was studied in nasal mucosa. Limited studies have indicated that IL-4 and IL-13 can induce, whereas INF-γ can inhibit the expression of SCGB3A1 (UGRP2) in a mouse transformed Clara cell line [22]; IL-5 may suppress SCGB3A2 (UGRP1) expression in murine lung tissues *in vivo *[23]; and INF-γ can up-regulate SCGB1D4 (IIS) expression in human lymphoblast cells [3]. In the present study, we found that SCGBs expression in human nasal mucosa could be differentially modulated by various inflammatory cytokines. On the other hand, the same cytokine could evoke distinct responses of different SCGB genes. Our results suggest that the expression of SCGBs is finely controlled by local immune responses in airways and SCGBs may be involved in cytokine-induced airway inflammation.

In present study, we found altered expression profiles of SCGBs in CRS. Since the biological functions of SCGBs are poorly understood, their roles in CRS mostly remain speculative. The unique increase of SCGB1C1 (RYD5) and decrease of SCGB3A1 (UGRP2) in CRSwNP, but not in CRSsNP, suggest that these two SCGBs may be more particularly involved in the polyp formation. Choi et al found that SCGB1D4 (IIS) can modulate lymphoblast cell migration [3]; therefore, the enhanced expression of SCGB1D4 (IIS) in both CRSsNP and CRSwNP may contribute to the persistent immune responses in CRS. The expression of SCGB1D1 (LIPA), SCGB2A1 (MGB2), and SCGB2A2 (MGB1) has been shown to be upregulated in human lung cancers [1,2]. In the current study, we found that SCGB2A2 (MGB1) expression was upregulated, whereas SCGB2A1 (MGB2) expression was down-regulated in both CRSsNP and CRSwNP, which is consistent with our previous gene array data [6]. In addition, we found that SCGB1D1 (LIPA) expression was up-regulated in CRSsNP. The aberrant expression of these three SCGBs suggests that they may be related to epithelial proliferation and tissue hyperplasia in CRS. However, obviously, further studies are needed to test these hypotheses and to elucidate the roles of SCGBs in CRS.

Among SCGBs, SCGB3A2 (UGRP1) is particularly interesting, given its structure and function similarities with SCGB1A1 (CC10) [4,9]. SCGB3A2 (UGRP1) is highly specific for airways [4,9]. Although the decreased expression and an anti-inflammatory role of SCGB3A2 (UGRP1) in allergic airway inflammation was observed in an animal model [4,9], its involvement in human airway diseases is largely unknown. In this study, we found that the mRNA and protein expression of SCGB3A2 (UGRP1) was dramatically down-regulated in both CRSsNP and CRSwNP, and SCGB3A2 (UGRP1) was mainly produced by epithelial cells in sinonasal mucosa, which is similar to the expression pattern of SCGB1A1 (CC10) [5-7]. Contrast to our findings, Burbure et al found that SCGB3A2 (UGRP1) levels in sputum were increased in patients with asthma and rhinitis [24]. However, they did not examine the SCGB3A2 (UGRP1) expression in local mucosa and the protein levels in sputum may not correlate with the expression intensity in local mucosa. As shown in our current study, SCGB3A2 (UGRP1) expression could be modulated by proinflammatory, Th1 and Th2 cytokines in nasal mucosa; therefore, the decreased expression of SCGB3A2 (UGRP1) in CRS might relate to the specific local cytokine environment in CRS [5,25]. More importantly, we found significant negative correlations between SCGB3A2 (UGRP1) expression and total inflammatory cells infiltration and disease severity evaluated by CT scan. In the light of the potential anti-inflammatory function of SCGB3A2 (UGRP1) indicated by animal experiments, our results suggest that the loss of SCGB3A2 (UGRP1) expression may contribute to the hyper-inflammation in CRS. However, this needs to be validated by function studies in human subjects in future. Furthermore, we found that SCGB3A2 (UGRP1) levels negatively correlated with post-operative symptom and endoscopy scores. This would be of considerable clinical value. Since detecting of potential responders among candidates for surgical treatment by certain biomarkers remains a difficult task [26]. This finding not only suggests that SCGB3A2 (UGRP1) may be a predictor of surgical response but also strengthens the involvement of SCGB3A2 (UGRP1) in the pathogenesis of CRS.

In this study, inferior turbinates were used as control, because it is difficult to obtain enough normal ethmoid mucosa samples due to ethical consideration. It is known that sinus and turbinate mucosa are both covered by the respiratory epithelium and share a number of similarities in histology and expression profiles of many common and important immune and biological molecules. Although we are not able to rule out the possibility that the differences observed between controls and CRS might be influenced by comparing these different tissue localizations, one could see clear differences between ethmoid tissue obtained from CRSsNP and CRSwNP patients.

In conclusion, this study, for the first time, shows the expression features of SCGB superfamily members in CRS and their cytokine-driven regulation in upper airways. Our results suggest the reduced production of SCGB3A2 (UGRP1), which is likely due, at least in part, to a local inflammatory environment, may contribute to the hyperinflammation in CRS and correlates with response to surgery.

## Competing interests

The authors declare that they have no competing interests.

## Authors' contributions

All authors read and approved the final manuscript. XL performed PCR experiments and data analysis. NW performed data analysis and manuscript preparation. XBL did immunohistochemical staining. XJY and YHC participated in tissue sample collection and some experiments. ZL designed the study and prepared the manuscript.

## References

[B1] Zafrakas M Petschke B Donner A Fritzsche F Kristiansen G Knuchel R Dahl E Expression analysis of mammaglobin A (SCGB2A2) and lipophilin B (SCGB1D2) in more than 300 human tumors and matching normal tissues reveals their co-expression in gynecologic malignancies BMC Cancer 2006 6 88 10.1186/1471-2407-6-88 16603086 PMC1513245

[B2] Sjödin A Guo D Sørhaug S Bjermer L Henriksson R Hedman H Dysregulated secretoglobin expression in human lung cancer Lung Cancer 2003 41 49 56 12826312 10.1016/s0169-5002(03)00126-0

[B3] Choi MS Ray R Zhang Z Mukherjee AB INF-gamma stimulates the expression of a novel secretoglobin that regulates chemotactic cell migration and invasion J Immunol 2004 172 4245 4252 15034037 10.4049/jimmunol.172.7.4245

[B4] Niimi T Keck-Waggoner CL Popescu NC Zhou Y Levitt RC Kimura S UGRP1, an uteroglobin/Clara cell secretory protein-related protein, is a novel lung-enriched downstream target gene for the T/EBP/NKX2.1 homeodomain transcription factor Mol Endocrinol 2001 15 2021 2036 10.1210/me.15.11.2021 11682631

[B5] Liu Z Lu X Zhang XH Bochner BS Long XB Zhang F Wang H Cui YH Clara cell 10-kDa protein expression in chronic rhinosinusitis and its cytokine-driven regulation in sinonasal mucosa Allergy 2009 64 149 157 10.1111/j.1398-9995.2008.01847.x 19076932

[B6] Liu Z Kim J Sypek JP Wang IM Horton H Oppenheim FG Bochner BS Gene expression profiles in human nasal polyp tissues studied by means of DNA microarray J Allergy Clin Immunol 2004 114 783 790 10.1016/j.jaci.2004.04.052 15480316

[B7] Wang H Long XB Cao PP Wang N Liu Y Cui YH Huang SK Liu Z Clara cell 10-kD protein suppresses chitinase 3-like 1 expression associated with eosinophilic chronic rhinosinusitis Am J Respir Crit Care Med 2010 181 908 916 10.1164/rccm.200904-0597OC 20093645 PMC2862304

[B8] Shijubo N Itoh Y Yamaguchi T Imada A Hirasawa M Yamada T Kawai T Abe S Clara cell protein-positive epithelial cells are reduced in small airways of asthmatics Am J Respir Crit Care Med 1999 160 930 933 10471621 10.1164/ajrccm.160.3.9803113

[B9] Chiba Y Kurotani R Kusakabe T Miura T Link BW Misawa M Kimura S Uteroglobin-related protein 1 expression suppresses allergic airway inflammation in mice Am J Respir Crit Care Med 2006 173 958 964 10.1164/rccm.200503-456OC 16456148 PMC2582904

[B10] Inoue K Wang X Saito J Tanino Y Ishida T Iwaki D Fujita T Kimura S Munakata M Plasma UGRP1 levels associate with promoter G-112A polymorphism and the severity of asthma Allergol Int 2008 57 57 64 10.2332/allergolint.O-07-493 18089940 PMC2768603

[B11] Niimi T Munakata M Keck-Waggoner CL Popescu NC Levitt RC Hisada M Kimura S A polymorphism in the human UGRP1 gene promoter that regulates transcription is associated with an increased risk of asthma Am J Hum Genet 2002 70 718 725 10.1086/339272 11813133 PMC384948

[B12] Meltzer EO Hamilos DL Hadley JA Lanza DC Marple BF Nicklas RA Adinoff AD Bachert C Borish L Chinchilli VM Danzig MR Ferguson BJ Fokkens WJ Jenkins SG Lund VJ Mafee MF Naclerio RM Pawankar R Ponikau JU Schubert MS Slavin RG Stewart MG Togias A Wald ER Winther B Rhinosinusitis Initiative: Rhinosinusitis: establishing definitions for clinical research and patient care J Allergy Clin Immunol 2004 114 s155 s212 10.1016/j.jaci.2004.09.029 17084217

[B13] Fokkens W Lund V Mullol J European Position Paper on Rhinosinusitis and Nasal Polyps Group European position paper on rhinosinusitis and nasal polyps 2007 Rhinol Suppl 2007 20 1 136 17844873

[B14] Lund VJ Kennedy DW Staging for rhinosinusitis Otolaryngol Head Neck Surg 1997 117 S35 40 10.1016/S0194-5998(97)70005-6 9334786

[B15] Lanza DC Kennedy DW Adult rhinosinusitis defined Otolaryngol Head Neck Surg 1997 117 3 Pt 2 s1 s7 10.1016/S0194-5998(97)70001-9 9334782

[B16] Liu Z Lu X Wang H You XJ Gao QX Cui YH Group II subfamily secretory phospholipase A2 enzymes: expression in chronic rhinosinusitis with and without nasal polyps Allergy 2007 62 999 1006 10.1111/j.1398-9995.2007.01381.x 17578498

[B17] Hauber HP Daigneault P Frenkiel S Lavigne F Hung HL Levitt RC Hamid Q Niflumic acid and MSI-2216 reduce TNF-α-induced mucin expression in human airway mucosa J Allergy Clin Immunol 2005 115 266 271 10.1016/j.jaci.2004.09.039 15696080

[B18] Benson M Carlsson L Adner M Jernas M Rudemo M Sjogren A Svensson PA Uddman R Cardell Lo: Gene profiling reveals increased expression of uteroglobin and other anti-inflammatory genes in glucocorticoid-treated nasal polyps J Allergy Clin Immunol 2004 113 1137 1143 10.1016/j.jaci.2004.02.028 15208596

[B19] Bin LH Nielson LD Liu X Mason RJ Shu HB Identification of uteroglobin-related protein 1 and macrophage scavenger receptor with collagenous structure as a lung-specific ligand-receptor pair J Immunol 2003 171 924 930 12847263 10.4049/jimmunol.171.2.924

[B20] Niimi T Copeland NG Gilbert DJ Jenkins NA Srisodsai A Zimonjic DB Keck-Waggoner CL Popescu NC Kimura S Cloning, expression, and chromosomal location of the mouse gene (Scgb3a1, alias Ugrp2) that encodes a member of the novel uteroglobin-related protein gene family Cytogenet Genome Re 2002 97 120 127 10.1159/000064067 12438750

[B21] Dear TN Boehm T Keverne EB Rabbitts TH Novel genes for potential ligand-binding protein in subregions of the olfactory mucosa The EMBO Journal 1991 10 2813 2819 1915264 10.1002/j.1460-2075.1991.tb07830.x PMC452990

[B22] Yamada A Sheikh F Niimi T DeMayo FJ Keegan AD Donnelly RP Kimura S Induction of uteroglobin-related protein 2 (UGRP2) gene expression by the Th2 cytokines IL-4 and IL-13 J Immunol 2005 175 5708 5715 16237061 10.4049/jimmunol.175.9.5708 PMC1364478

[B23] Chiba Y Srisodsai A Supavilai P Kimura S Interleukin-5 reduces the expression of uteroglobin-related protein (UGRP) 1 gene in allergic airway inflammation Immunol Lett 2005 97 123 129 10.1016/j.imlet.2004.10.013 15626484 PMC1343456

[B24] de Burbure C Pignatti P Corradi M Malerba M Clippe A Dumont X Moscato G Mutti A Bernard A Uteroglobin-related protein 1 and clara cell protein in induced sputum of patients with asthma and rhinitis Chest 2007 131 172 179 10.1378/chest.06-0835 17218572

[B25] Cao PP Li HB Wang BF Wang SB You XJ Cui YH Wang DY Desrosiers M Liu Z Distinct immunopathologic characteristics of various types of chronic rhinosinusitis in adult Chinese J Allergy Clin Immunol 2009 124 478 484 10.1016/j.jaci.2009.05.017 19541359

[B26] Lane AP Truong-Tran QA Schleimer RP Altered expression of genes associated with innate immunity and inflammation in recalcitrant rhinosinusitis with polyps Am J Rhinol 2006 20 138 144 16686375 PMC2810150

